# A Rare Case of Vanishing Lung Syndrome

**DOI:** 10.1155/2011/957463

**Published:** 2011-10-09

**Authors:** Nidhi Sood, Nikhil Sood

**Affiliations:** ^1^Department of Internal Medicine, University of Missouri, One Hospital Drive, Columbia, MO 65212, USA; ^2^Department of Internal Medicine, Winthrop University Hospital, 200 Old Country Road, Mineola, NY 11501, USA

## Abstract

We describe here a rare case of Idiopathic Bullous Emphysema/Vanishing Lung Syndrome (VLS) in a 33-year-old male patient with a history of marijuana abuse who presents to the hospital with pleuritic chest pain thought to be due to pneumothorax based on the chest radiograph. This case emphasizes the need to obtain chest computed tomography in a relatively stable patient suspected of VLS to reduce the potential risk of overseeing a bronchopleural fistula.

## 1. Case Presentation

**Figure 1:**
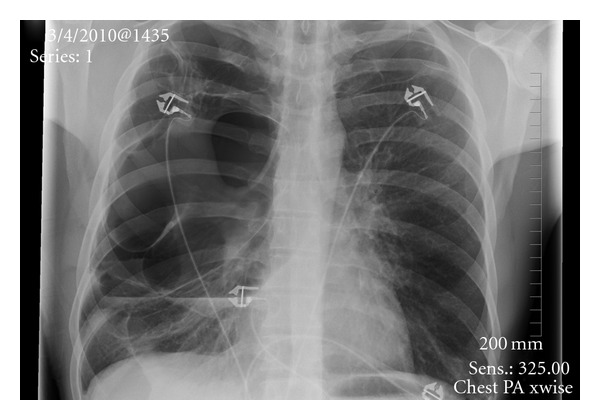
Chest X ray showing bullous disease.

**Figure 2:**
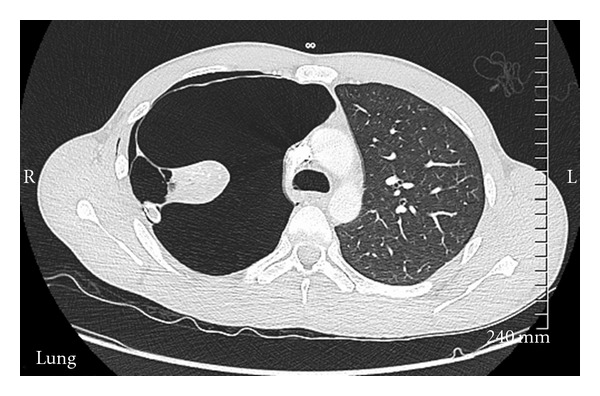
Chest CT showing bullous disease.

**Figure 3:**
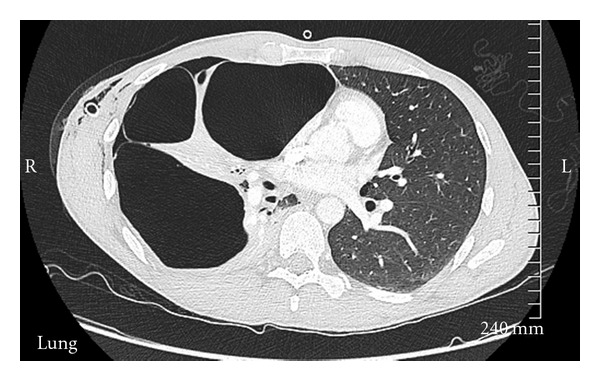
Chest CT at different section showing bullous disease.

 Our patient is a 33-year-old male who was admitted with complaints of shortness of breath. Patient denied any fever, trauma. He was initially hypoxic and required 3 liters of oxygen. Patient's past medical history was positive for tobacco abuse, 1 pack per day over past 15 years, Marijuana abuse off and on past 10 years, and he was working as a handyman. Initially laboratory tests including complete blood count with differential, complete metabolic profile, cardiac enzymes, brain natriuretic peptide, and electrocardiogram were within normal limits. The initial arterial blood gas analysis on three litres oxygen showed ph 7.47, po2 150, and pco2 38. Chest X-ray done revealed large bullous lesion on both sides with concern for right pneumothorax (Figure 1). A chest tube was placed but the chest X-ray remained unchanged. Chest X-ray had shown large bullous lesion mistaken as pneumothorax (Figure 1). CT of the chest revealed the diagnosis of giant bullous emphysema (GBE) (Figures 2 and 3). Urine toxicology was negative. With alpha 1 antitrypsin studies being negative, it was concluded as case of vanishing lung syndrome (VLS)/Idiopathic giant bullous emphysema with known traditional risk factors of tobacco and marijuana abuse. The patient was discharged three days later on room air with plan of completing pulmonary function testing and outpatient pulmonary rehabilitation with possibility of bullectomy.

## 2. Discussion

 A distinct clinical syndrome, Giant Bullous Emphysema or VLS, a primary bullous disease of the lung, or Type I bullous disease is defined as a large bulla occupying at least one-third of a hemithorax [1–3].

Risk factors include smoking, alpha 1 antitrypsin deficiency, and marijuana abuse [4–6]. Marijuana smoking leads to asymmetrical bullous disease, often in the setting of normal chest X-ray and lung function. In subjects who smoke marijuana, these pathological changes occur at a younger age (approximately 20 years earlier) than in tobacco smokers [4, 5].

The radiographic criteria for vanishing lung syndrome, as defined by Roberts and colleagues [2], include the presence of giant bulla in one or both upper lobes, occupying at least one third of the hemithorax and compressing surrounding normal lung parenchyma [2]. A major complication of VLS is pneumothorax, which classically involves a history of acute deterioration in respiratory function associated with chest pain. Also infection of the bullae is common [7]. High resolution computerized tomography (HRCT) is used for preoperative assessment and shows the extent and distribution of the bullous disease to accurately determine the possible cause of the symptoms. HRCT also allows assessment of coexisting conditions such as infected cysts, bronchiectasis, pulmonary artery enlargement, and pneumothorax [8].

Determination of the preoperative bulla volume allows the prediction of the expected increase of postoperative FEV1. Bullectomy causes significant improvements in dyspnea, gas exchange, pulmonary function, and exercise capacity, with the best results being obtained in the more significant VLS cases. On average, improvements persist for approximately 3 to 4 years but begin to decline thereafter [9].

Giant bullectomy is shown to produce significant immediate functional improvement. This benefit declines with time but persists at least 3 years. This based on the followup of 43 patients with giant bullous emphysema who were followed up for a mean duration of 4.5 years [10]. There was significant improvement in all three measurements of FVC, FEV, and dyspnea grading in the early postoperative period, but at 5–10 years only the FVC improvement was significant [11].

## 3. Conclusion

 Vanishing Lung Syndrome is a rare condition which becomes clinically evident in a much advanced stage. Patients should be strongly counseled against any further tobacco and marijuana abuse. These patients should be referred to pulmonology and cardiothoracic surgery to further delineate plans for surgery at the appropriate time.
